# Hyperkyphosis and self-reported and objectively measured sleep quality in older men

**DOI:** 10.1371/journal.pone.0228638

**Published:** 2020-02-11

**Authors:** Christopher N. Kaufmann, Jian Shen, Gina N. Woods, Nancy E. Lane, Katie L. Stone, Deborah M. Kado, for the Osteoporotic Fractures in Men (MrOS) Study Research Group

**Affiliations:** 1 Division of Geriatrics and Gerontology, Department of Medicine, University of California San Diego School of Medicine, La Jolla, California, United States of America; 2 Division of Preventive Medicine, Department of Family Medicine and Public Health, University of California San Diego School of Medicine, La Jolla, California, United States of America; 3 Division of Endocrinology, Department of Medicine, University of California San Diego School of Medicine, La Jolla, California, United States of America; 4 Center for Musculoskeletal Health and Department of Internal Medicine, UC Davis, Sacramento, California, United States of America; 5 California Pacific Medical Center Research Institute, San Francisco, California, United States of America; McMaster University, CANADA

## Abstract

**Objectives:**

Hyperkyphosis is associated with restricted pulmonary function and posture, potentially contributing to poor sleep. A previous study reported older women with hyperkyphosis had worse self-reported sleep quality, but it is less clear if this association exists in men. We examined the association between hyperkyphosis and subjective and objective sleep quality in a cohort of older men.

**Design:**

Longitudinal analysis of data from large cohort of older men participating in the Osteoporotic Fractures in Men Study (MrOS).

**Setting:**

Community.

**Participants:**

We studied 754 men participants in MrOS who had kyphosis measured during the 3^rd^ clinic visit (2007–2009) and future subjective and objective sleep quality assessed between 2009–2012 (an average of 2.9 years later).

**Intervention:**

N/A.

**Measurements:**

To measure kyphosis, 1.7 cm thick wooden blocks were placed under the participant’s head to achieve a neutral spine position while lying supine on a DXA table. We collected data on both subjective (Pittsburgh Sleep Quality Index [PSQI], and Epworth Sleepiness Scale [ESS]) and objective (wrist actigraphy: Total Sleep Time [TST], Wake After Sleep Onset [WASO], Sleep Efficiency [SE], Sleep Onset Latency [SOL]; and polysomnography: Apnea Hypopnea Index [AHI]) sleep measurements. Those who required >3 blocks were considered hyperkyphotic (n = 145 or 19.2%).

**Results:**

In unadjusted and multivariable analyses, men with hyperkyphosis did not report having worse self-reported sleep characteristics based on PSQI and ESS. Similarly, there were no significant associations between hyperkyphosis and objective sleep measures. When examined as a continuous predictor (blocks ranging from 0–8), results were no different.

**Conclusions:**

Although we hypothesized that poor posture in those with hyperkyphosis would interfere with sleep, in this sample of older men, worse kyphosis was not associated with self-reported or objectively measured poor sleep quality.

## Introduction

Hyperkyphosis, the excessive anterior curvature of the thoracic spine, affects an estimated 20–40 percent of older people [1–3]. Despite being relatively common in the population with no standard diagnoses or treatments available, hyperkyphosis is associated with a number of serious health complications, including physical disability [4], increased risk of fractures [5], and even premature mortality [6]. As hyperkyphosis progresses with advancing age, there is a need for better understanding of the extent to which it may affect daily functioning needed for healthy aging.

Hyperkyphosis is also associated with restricted pulmonary function [7] and posture [8], which may impact sleep quality possibly by increasing sleep fragmentation. Additionally, available treatments for hyperkyphosis, including physical therapy, therapeutic exercises, use of bracing, and surgery [9], may all impact sleep as well. Surprisingly, there has only been one study examining the association between hyperkyphosis and sleep quality [10], and this study was cross-sectional. Wankie and colleagues showed that older women with hyperkyphosis had worse self-reported sleep quality whereas no association was found in men [10]. While there was no association in men, the measure of sleep was subjective, and it is possible that the impact of hyperkyphosis in men might be better captured by objectively measured sleep dysfunction. Additionally, given the importance of sleep for healthy aging, it is important to explore whether relationships of hyperkyphosis with sleep be examined longitudinally to determine whether individuals with hyperkyphosis are susceptible to developing sleep problems over time.

To address these gaps, we analyzed the longitudinal association between hyperkyphosis and future sleep quality among older men participating in the Osteoporotic Fractures in Men Study (MrOS), using both subjective and objective sleep quality measures. The specific aims of our analysis were to determine whether there were differences in a) subjective and b) objective measurements of sleep quality between those with hyperkyphosis and those without. We hypothesized that hyperkyphosis will be associated with impairments in both subjective and objective sleep quality in older men.

## Methods

### Data source

Data for this study came from the Osteoporotic Fractures in Men Study (MrOS), a multi-center longitudinal observational cohort study of older men. From 2000–2002, 5,994 community-dwelling men age 65+ years were recruited from six sites in the US: Birmingham, Alabama; Minneapolis, Minnesota; Palo Alto, California; Monongahela Valley, Pennsylvania; Portland, Oregon; and San Diego, California. Eligibility requirements for study participation included walking without assistance and no history of bilateral hip replacement. From 2003–2005, a total of 3,135 MrOS participants were recruited to participate in the MrOS Sleep Study, an ancillary study which sought to characterize subjective and objective sleep quality among participants. For MrOS Sleep, participants were screened for nightly (or near nightly) use of mechanical devices (e.g., CPAP or BiPAP for sleep apnea, supplemental oxygen therapy), and were excluded from participation if they were unable or unwilling to forego use of such devices during the night of the sleep study. A second assessment in the sleep study was completed between 2009–2012, in which a subset of 1,055 original MrOS Sleep Study participants (recruitment goal was 1,000 subjects) repeated wrist actigraphy. To date, MrOS parent study has had four visits spaced 3–4 years apart. Our study used hyperkyphosis data from the third MrOS parent study visit (fielded from March 2007-March 2009) and MrOS Sleep visit 2 (fielded from November 2009-March 2012). Mean follow-up time between these two visits is approximately 2.9 years (SD = 0.77). The design and conduct of MrOS and MrOS Sleep have been previously reported [11, 12]. All sites obtained their own institutional review board approval prior to completion of study (Sutter Health Institutional Review Board, University of California, San Francisco Human Research Protection Program, University of Alabama at Birmingham Institutional Review Board for Human Use, Human Research Protection Program at the University of Minnesota, Stanford University Institutional Review Board, University of Pittsburgh Institutional Review Board, Oregon Health & Science University Institutional Review Board, and the University of California, San Diego Human Research Protections Program). Additionally, data are publicly available at the following website: https://mrosdata.sfcc-cpmc.net/.

### Participants

**Fig 1:**
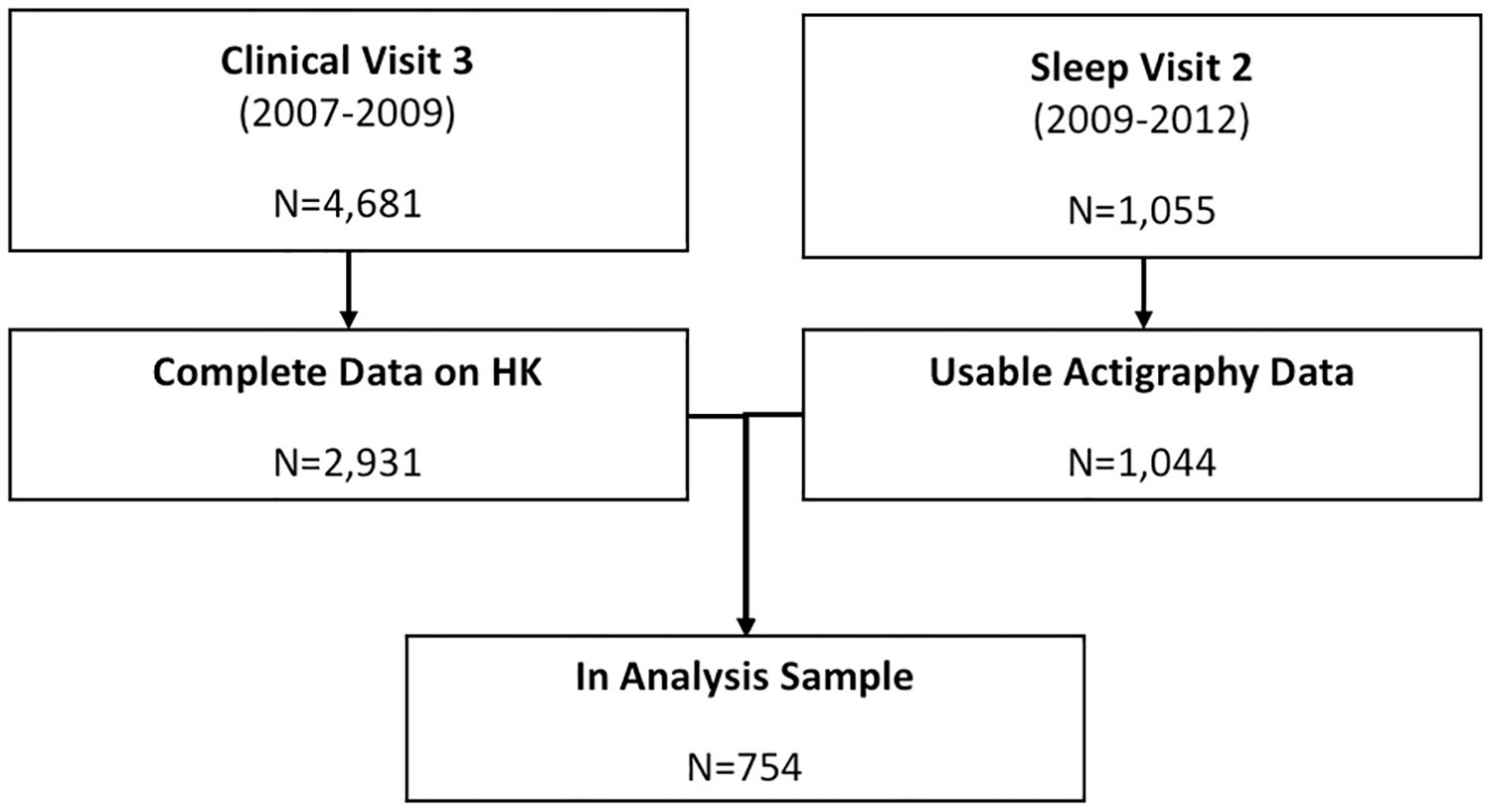
Flow diagram of analysis sample generation.

Participants in the MrOS Sleep Study who completed blocks measurement of kyphosis (described below) during MrOS visit 3, and had wrist actigraphy recorded at the MrOS Sleep visit 2, were included in analyses. Of the 1,044 subjects who provided actigraphy data for MrOS Sleep visit 2, 754 were among the 4,681 subjects who had also completed MrOS visit 3 and provided data for this analysis (See Fig 1). All MrOS study participants provided informed consent at each of the clinical centers to participate in the study. Institutional review board approval was obtained by institutions at each site.

### Measures

#### Hyperkyphosis

Measurement of hyperkyphosis was completed using the Rancho Bernardo block method [1]. Wooden blocks of 1.7 cm thickness were placed under the participant’s head to achieve a neutral spine position while lying supine on a DXA table. The number of blocks needed to yield a neutral spine (e.g., head is not hyperextended or hyperflexed) was recorded. Interrater reliability of the block method for measuring hyperkyphosis has ranged from 0.85 to 1.00 Spearman correlation across clinical sites. This study defined presence of hyperkyphosis as >3 blocks.

#### Subjective sleep quality

Self-reported sleep quality measures included the Pittsburgh Sleep Quality Index (PSQI) [13] and the Epworth Sleepiness Scale (ESS) [14]. The PSQI measures self-reported sleep quality and sleep disturbances over the past month, and consists of a global score (with scores ranging from 0 to 21; scores >5 are considered as poor sleep quality), as well as seven component scores including subjective quality, latency, duration, efficiency, sleep disturbance, sleep medication usage, and daytime dysfunction (scores range from 0 to 3 for each component). The ESS is a questionnaire that measures subjective daytime sleepiness with scores ranging from 0 to 24 (scores >10 are considered as excessive daytime sleepiness). For the PSQI and ESS, higher scores indicate worse symptomatology.

#### Objective sleep quality

Objective sleep quality was measured through the use of wrist actigraphy, a validated method [15] to estimate sleep/wake patterns based upon movement detected from a wrist worn device called an actigraph. Specifically, the actigraph model used was the Actiwatch 2 (Respironics, Inc., Bend, Oregon). Subjects were asked to wear the actigraph around the non-dominant wrist for five consecutive days, only taking the device off for bathing and while swimming. Participants on average wore the watch for a mean of 5.0 days (SD = 0.73, Range = 1–14 days). Movement data are recorded continuously throughout the day and summed in 1-minute intervals (epochs) with a 20 count threshold for determination of sleep/wake categorization. Concurrently with wrist actigraphy, participants were asked to self-report the times they went into and out of bed, when they turned lights out, as well as any periods in which the actigraph was not worn. Trained scorers identify intervals during which the device was removed, and mark the night-time sleep period with assistance from this diary. Action W-2 software was used to classify each epoch as sleep or wake, and objective sleep variables were derived [16]. Variables estimated from wrist actigraphy during the night-time sleep period include total sleep time (TST; duration in minutes spent asleep), wake after sleep onset (WASO; time in minutes awake while in bed), sleep efficiency (SE; percentage of time in bed spent asleep), and sleep latency (SL; time to fall asleep after lights out). All variables were averaged over all nights of data collection.

#### Sleep apnea

In addition to wrist actigraphy, subjects underwent a single night, unattended in-home polysomnography session. Methods have been previously described [17]. AHI was defined as number of apneas or hypopneas per hour of sleep with at least 3% oxygen desaturation. An apnea was defined as a complete cessation of breathing, where as a hypopnea was a partial (at least 50% reduction in airflow), each lasting at least 10 seconds.

#### Covariates

We also examined a number of covariates including baseline age, height, weight, marital status, self-reported health status, physical activity as measured by the Physical Activity Scale for the Elderly (PASE) [18], smoking status, history of alcohol use, and selected medical conditions (including sleep apnea, depression, arthritis, cardiovascular disease, diabetes). Additionally, via reports from clinics, MrOS collected data on prescription medications used of which were coded using the Iowa Drug Information Service (IDIS) Drug Vocabulary (College of Pharmacy, University of Iowa, Iowa City, IA) [19]. We assessed sleep medications, antidepressants, and benzodiazepines.

### Analyses

Baseline characteristics of participants with vs. without hyperkyphosis were compared using chi-squared tests for binary/categorical variables and F-tests for continuous variables. In order to compare those with and without hyperkyphosis, we used linear regression models to analyze the associations between baseline hyperkyphosis and future subjective (PSQI total and component scores and ESS) and objective (TST, WASO, SE, and SL from wrist actigraphy; RDI at 3% desaturation from overnight in-home polysomnography) sleep outcomes. We further used logistic regression to determine the association between hyperkyphosis and dichotomous sleep outcomes, based on established cut points to define those with poor sleep. We conducted both bivariate and multivariate analyses, adjusting for significant baseline characteristics associated with hyperkyphosis at *p*<0.10 and study site. All analyses were completed in Stata SE version 15 (Stata Corp, College Station, TX).

## Results

Subject characteristics by hyperkyphosis group are displayed in Table 1. Hyperkyphosis subjects were on average 79.5 years old (SD = 5.30), had a height of 174.9 cm (SD = 7.84) and weight of 85.3 kg (SD = 13.93). Ninety percent were Caucasian, 71.7% were married, and 88.3% were in excellent or good health status. The mean PASE score was 128.0 (SD = 64.08). Fewer than 1% were current smokers, and subjects reported an average of 1.8 drinks per week (SD = 1.60). In terms of health conditions, 16.2% reported sleep apnea, 11.7% depression, 6.2% arthritis, 73.1% cardiovascular disease, and 17.2% diabetes. For medication use, just under a fifth reported use of sleep medications and similar percentages were reported for antidepressant use. Four-percent reported use of benzodiazepines. Compared to the non-hyperkyphosis group, the hyperkyphosis group was older, taller and heavier, less likely to be married, less physically active and more likely to have cardiovascular disease (Table 1).

**Table 1:** Baseline characteristics.

	Hyperkyphosis		Comparison
	Normal n = 609 n (%)^1^	Hyperkyphotic n = 145 n (%)^1^	*p*-value^2^
**Age (years, mean ± SD)**	77.6 (4.53)	79.5 (5.30)	**<0.001**
**Height (cm, mean ± SD)**	173.5 (6.68)	174.9 (7.84)	**0.027**
**Weight (kg, mean ± SD)**	81.0 (12.73)	85.3 (13.93)	**<0.001**
**Race/Ethnicity**			0.105
White	508 (83.4)	130 (89.7)	
African American	32 (5.3)	2 (1.4)	
Asian	41 (6.7)	5 (3.5)	
Hispanic	16 (2.6)	6 (4.1)	
Other	12 (2.0)	2 (1.4)	
**Marital Status**			**0.029**
Married	496 (81.4)	104 (71.7)	
Widowed	63 (10.3)	29 (20.0)	
Separated	2 (0.3)	1 (0.7)	
Divorced	29 (4.8)	7 (4.8)	
Single, never married	19 (3.1)	4 (2.8)	
**Self-Reported Health Status**			0.814
Fair/poor or very poor	67 (11.0)	17 (11.7)	
Excellent or good	540 (89.0)	128 (88.3)	
**Physical Activity** **(PASE score, mean ± SD)**	145.8 (67.36)	128.0 (64.08)	**0.004**
**Smoking Status**			0.732
No	263 (43.3)	65 (44.8)	
Past	335 (55.2)	79 (54.5)	
Current	9 (1.5)	1 (0.7)	
**Alcohol Use** **(drinks/wk, mean ± SD)**	1.9 (1.71)	1.8 (1.60)	0.480
**Selected Medical Conditions**^3^			
Sleep apnea	78 (13.2)	23 (16.2)	0.352
Depression	44 (7.2)	17 (11.7)	0.074
Arthritis	44 (7.2)	9 (6.2)	0.666
Cardiovascular disease	375 (61.6)	106 (73.1)	**0.009**
Diabetes	89 (14.6)	25 (17.2)	0.427
**Selected medication use**^3^			
Sleep medication	82 (13.5)	24 (16.6)	0.340
Antidepressant	57 (9.4)	21 (14.5)	0.070
Benzodiazepine	23 (3.8)	5 (3.5)	0.848
**Study site**			**<0.001**
Birmingham, AL	101 (16.6)	22 (15.2)	
Minneapolis, MN	85 (14.0)	19 (13.1)	
Palo Alto, CA	79 (13.0)	50 (34.5)	
Monongahela Valley, PA	111 (18.2)	11 (7.6)	
Portland, OR	101 (16.6)	30 (20.7)	
San Diego, CA	132 (21.7)	13 (9.0)	

^1^ All cells report column percentages unless otherwise specified

^2^ *p*-value corresponds to chi-square tests for binary/categorical variables, and F-test for continuous variables

^3^ Column percentages for medical conditions and medications do not add to 100%; each condition/medication is measured dichotomously

No significant differences between the hyperkyphosis and non-hyperkyphosis groups were observed for all subjective (PSQI, ESS) and objective (wrist actigraphy and polysomnography) sleep measures in both unadjusted and adjusted models (Tables 2 and 3). In examining hyperkyphosis as a continuous measure (e.g., number of blocks with scores from 0–8 blocks), we noted no statistically significant correlations between severity of hyperkyphosis and sleep measures (PSQI: B = 0.05, 95% CI = -0.11, 0.22; ESS: B = 0.10, 95% CI = -0.11, 0.31; TST: B = 2.31, 95% CI = -1.23, 5.84; WASO: -0.42, 95% CI = -2.11, 1.27; SE: B = 0.09, 95% CI = -0.28, 0.46; SOL: B = 1.10, 95% CI = -0.81, 3.02; AHI: B = -0.25, 95% CI = -1.11, 0.62). Additionally, using commonly used cut points for subjective and objective sleep quality (i.e., PSQI>5, ESS≥10, actigraphic TST<5 hours, SE<80%, WASO>90 minutes, SOL>30 minutes, and AHI>15), we also observed no associations with hyperkyphosis as the predictor (PSQI: OR = 1.15, 95% CI = 0.80, 1.66; ESS: OR = 1.23, 95% CI = 0.82, 1.85; TST: OR = 0.94, 95% CI = 0.45, 1.99; WASO: OR = 1.26, 95% CI = 0.85, 1.85; SE: OR = 1.06, 95% CI = 0.72, 1.57; SOL: OR = 1.09, 95% CI = 0.76, 1.57; AHI: OR = 1.15, 95% CI = 0.80, 1.66). Given that long-duration sleep (in addition to short-duration sleep) may also be associated with poor health outcomes [20], we repeated the cut point analysis for TST but excluded those with >8 hours sleep, and results were still non-significant (OR = 0.99, 95% CI = 0.47, 2.10).

**Table 2:** Subjectively measured sleep quality by hyperkyphosis status.

	Hyperkyphosis		Comparison	
	Normal Mean (SD)	Hyperkyphotic Mean (SD)	Bivariate β (95% CI)^1^	Multivariate β (95% CI)^1^^,^^2^
**PSQI**				
Global score	5.4 (3.04)	5.7 (2.98)	0.26 (-0.29, 0.81)	0.10 (-0.48, 0.68)
Subjective quality	0.8 (0.69)	0.8 (0.64)	0.01 (-0.11, 0.13)	-0.01 (-0.15, 0.12)
Latency	0.8 (0.85)	0.9 (0.92)	0.13 (-0.02, 0.29)	0.07 (-0.09, 0.24)
Duration	0.7 (0.64)	0.6 (0.62)	-0.10 (-0.22, 0.01)	-0.03 (-0.15, 0.09)
Efficiency	0.6 (0.87)	0.6 (0.89)	0.05 (-0.11, 0.21)	0.05 (-0.12, 0.22)
Disturbance	1.3 (0.54)	1.2 (0.48)	-0.05 (-0.15, 0.04)	-0.08 (-0.19, 0.02)
Sleep medication	0.5 (1.03)	0.7 (1.17)	0.16 (-0.03, 0.35)	0.13 (-0.07, 0.34)
Daytime dysfunction	0.7 (0.68)	0.8 (0.63)	0.05 (-0.07, 0.18)	-0.04 (-0.16, 0.09)
**ESS**	6.8 (3.93)	7.3 (4.17)	0.50 (-0.22, 1.22)	-0.01 (-0.77, 0.75)

**Notes:** SD = Standard Deviation; β = Beta Coefficient; 95% CI = 95% Confidence Interval; PSQI = Pittsburgh Sleep Quality Index; ESS = Epworth Sleepiness Scale

^1^ Corresponds to beta coefficient in linear regression models for the difference in subjective sleep measures by kyphosis severity groups.

^2^ Models are adjusted for age, height, weight, marital status, PASE score, cardiovascular disease, and study site.

**Table 3:** Objectively measured sleep quality by hyperkyphosis status.

	Hyperkyphosis		Comparison	
	Normal Mean (SD)	Hyperkyphotic Mean (SD)	Bivariate β (95% CI)^1^	Multivariate β (95% CI)^1^^,^^2^
**Total Sleep Time, minutes**	393.5 (65.95)	398.3 (67.60)	4.78 (-7.25, 16.80)	-3.53 (-16.15, 9.09)
**WASO, minutes**	78.0 (32.27)	78.9 (28.87)	0.90 (-4.84, 6.64)	-0.29 (-6.35, 5.78)
**Sleep Efficiency, %**	82.7 (7.00)	82.5 (6.77)	-0.19 (-1.45, 1.07)	-0.20 (-1.54, 1.15)
**Sleep Latency, minutes**	38.7 (33.73)	41.9 (44.05)	3.21 (-3.30, 9.73)	3.00 (-4.04, 10.04)
**Apnea Hypopnea Index**	18.5 (15.94)	19.4 (17.11)	0.98 (-2.00, 3.95)	0.95 (-2.17, 4.08)

**Notes:** SD = Standard Deviation; β = Beta Coefficient; 95% CI = 95% Confidence Interval; WASO = Wake After Sleep Onset

^1^ Corresponds to beta coefficient in linear regression models for the difference in objective sleep measures by kyphosis severity groups.

^2^ Models are adjusted for age, height, weight, marital status, PASE score, cardiovascular disease, and study site.

Finally, as participation in MrOS Sleep visit 2 required that a participant not use a respiratory device at night (e.g., CPAP) or other oxygen treatment, and may thus have excluded those with the most severe sleep disturbances, we also conducted a sensitivity analysis to determine whether hyperkyphosis was associated with subjective sleep quality (as measured by the PSQI) administered to the entire MrOS cohort at Visit 4 where this exclusion criteria was not applied. Among the individuals with complete data for hyperkyphosis at Visit 3 and PSQI data for Visit 4 an average of 7.4 (SD = 0.57) years later (N = 1,607), we found unadjusted associations between hyperkyphosis and the PSQI components of sleep duration (B = -0.08, 95% CI = -0.17, 0.00) and daytime dysfunction (B = 0.13, 95% CI = 0.05, 0.21). After adjusting for participant age at Visit 3, daytime dysfunction remained statistically significant (B = 0.12, 95% CI = 0.04, 0.20) (S1 Table).

## Discussion

In this population-based sample of community-dwelling older men, we hypothesized that presence of hyperkyphosis would be associated with poorer subjective and objective sleep quality. Our hypothesis was not supported, finding no differences in sleep measurements between those with and without hyperkyphosis. In addition, severity of hyperkyphosis was not associated with future sleep quality measures. These results are in line with findings from our previous study [10], and suggests that the pulmonary and postural complications seen in hyperkyphosis may not contribute to future poor sleep quality in men. As our previous study showed a significant cross-sectional relationship between hyperkyphosis and subjective sleep quality in women, replication of our longitudinal analysis in a sample of older women is needed.

Why sleep quality might be adversely affected by hyperkyphosis in women, but not men is not clear. The prevalence of hyperkyphosis is higher in women as compared to men, with underlying conditions contributing to hyperkyphosis (including osteoporosis, degenerative disk disease, spinal weaknesses, etc.) that are all more prevalent in women [21]. It is possible that these underlying conditions may be contributing to poor sleep rather than hyperkyphosis per se. Another explanation might be gender differences in postural changes with aging. In older women, the hyperkyphosis changes predominantly occur in the thoracic region while in men the cervical changes that would be captured by the blocks measure of kyphosis are much more prominent [1]. It is conceivable that thoracic versus cervically prominent hyperkyphosis would more adversely influence pulmonary function and therefore, measures of sleep quality. In our previous paper, the kyphosis measure used was the flexicurve that is placed along the thoracic and not cervical spine. It will be important for future studies to directly make comparisons between men and women.

More broadly from a clinical context, understanding the association between hyperkyphosis and sleep quality is important for determining factors contributing to hyperkyphosis prognosis and other aging outcomes over time. Sleep quality is well acknowledged as a major contributor to health and well-being across the entire life course, and obtaining proper sleep may help in minimizing the deleterious effects of hyperkyphosis and other underlying health conditions that are contributing to impairments in functioning. Additionally, by maintaining quality sleep, one might be able to improve the health outcomes of hyperkyphosis treatment, which may improve quality of life among those affected.

This study’s strengths include a large population-based sample with well-validated subjective and objective sleep measures and a longitudinal design. However, our study should be interpreted in the context of its limitations. First, wrist actigraphy was not administered to those using a respiratory device at night and who were not willing to discontinue use during sleep assessment. This may have resulted in a sample of participants with better pulmonary function and by proxy may have excluded those with the most severe sleep apnea (e.g., obstructive sleep apnea) limiting sleep quality. Thus, our null findings may be explained by having a sample of subjects who are less disabled by hyperkyphosis. Our sensitivity analyses suggest there were correlations between hyperkyphosis and some subjective PSQI sleep components, but this may be due to the larger sample size increasing power. Second, our results are only generalizable to older men. Additional longitudinal investigations are needed in women and more diverse samples. Third, we did not have data on treatment for hyperkyphosis. Forth, while we controlled for numerous confounding variables, residual confounding due to unmeasured confounders may be possible.

In summary, while we did not find an association between hyperkyphosis and subsequent sleep quality, more work is needed to determine the ways in which hyperkyphosis may influence not only sleep but other biological, behavioral and social factors important for healthy aging and prevention of disability. Such findings may substantially contribute to improving the health of an aging population.

## Supporting information

S1 Table Sensitivity analysis of Pittsburgh Sleep Quality Index scores at Visit 4 by hyperkyphosis status (N = 1,607). SD = Standard Deviation; b = Beta Coefficient; 95% CI = 95% Confidence Interval; PSQI = Pittsburgh Sleep Quality Index. ^1^Corresponds to beta coefficient in linear regression models for the difference in subjective sleep measures by kyphosis severity groups. ^2^Models are adjusted for age.
(DOCX)
