# Supplementary material for: Hyperkyphosis and self-reported and objectively measured sleep quality in older men
Source: PLoS One. 2020 Feb 11;15(2):e0228638. doi: 10.1371/journal.pone.0228638 (PMC7012394; doi:10.1371/journal.pone.0228638)
Supplement: S1 Table — SD = Standard Deviation; b = Beta Coefficient; 95% CI = 95% Confidence Interval; PSQI = Pittsburgh Sleep Quality Index. 1Corresponds to beta coefficient in linear regression models for the difference in subjective sleep measures by kyphosis severity groups. 2Models are adjusted for age. (DOCX) [file pone.0228638.s001.docx]

**S1 Table.** Sensitivity Analysis of Pittsburgh Sleep Quality Index Scores at Visit 4 by Hyperkyphosis Status (N=1,607)

|  | **Hyperkyphosis** | | **Comparison** | |
| --- | --- | --- | --- | --- |
|  | **Normal**  **Mean (SD)** | **Hyperkyphotic**  **Mean (SD)** | **Bivariate**  β **(95% CI)^1^** | **Adjusted for Age**  β **(95% CI)^1,2^** |
| **PSQI** |  |  |  |  |
| Global score | 5.4 (3.18) | 5.8 (3.13) | 0.38 (-0.02, 0.77) | 0.35 (-0.04, 0.75) |
| Subjective quality | 0.8 (0.65) | 0.8 (0.63) | 0.08 (0.00, 0.16) | 0.08 (0.00, 0.16) |
| Latency | 0.8 (0.89) | 0.9 (0.92) | 0.08 (-0.03, 0.19) | 0.07 (-0.05, 0.18) |
| Duration | 0.7 (0.67) | 0.6 (0.63) | **-0.08 (-0.17, 0.00)** | -0.07 (-0.16, 0.01) |
| Efficiency | 0.6 (0.92) | 0.7 (0.91) | 0.03 (-0.09, 0.14) | 0.02 (-0.10, 0.13) |
| Disturbance | 1.2 (0.50) | 1.3 (0.53) | 0.04 (-0.02, 0.10) | 0.04 (-0.02, 0.11) |
| Sleep medication | 0.6 (1.11) | 0.7 (1.16) | 0.11 (-0.03, 0.25) | 0.11 (-0.03, 0.25) |
| Daytime dysfunction | 0.8 (0.64) | 0.9 (0.65) | **0.13 (0.05, 0.21)** | **0.12 (0.04, 0.20)** |

**Notes:** SD = Standard Deviation; b = Beta Coefficient; 95% CI = 95% Confidence Interval; PSQI = Pittsburgh Sleep Quality Index

^1^ Corresponds to beta coefficient in linear regression models for the difference in subjective sleep measures by kyphosis severity groups

^2^ Models are adjusted for age
